# Convergence of the turkey gut microbiota following cohabitation under commercial settings

**DOI:** 10.1186/s40104-021-00580-4

**Published:** 2021-05-05

**Authors:** Elizabeth A. Miller, Brittanie Winfield, Bonnie P. Weber, Cristian Flores-Figueroa, Jeannette Munoz-Aguayo, Jared Huisinga, Timothy J. Johnson

**Affiliations:** 1 Department of Veterinary and Biomedical Sciences, University of Minnesota, Saint Paul, MN USA; 2 University of Minnesota, Mid-Central Research and Outreach Center, Willmar, MN USA; 3 Life Science Innovations, Willmar, MN USA

**Keywords:** Brood, Gut, Hatch, Microbiota, Poult, Succession, Turkey

## Abstract

**Background:**

Microbiota development is a critical aspect of turkey poult maturation, and the succession of microbes in the turkey gut has been shown to correlate with poult performance. The purpose of this study was to determine the fate of the microbiota in turkey poults after movement of birds first raised in an isolated hatch brood system into a more traditional commercial brood facility with pre-existing birds. Turkey poults were first divided into groups raised in conventional brood pens from day-of-hatch and those raised in an experimental hatch brood system. After 11 days of growth, hatch brood birds were moved into pens within the conventional brood barn and monitored for an additional 18 days. Sampling of both hatch brood and conventional pen birds was performed at multiple timepoints throughout the study, and cecal content was used to analyze the bacterial microbiota using 16S rRNA gene amplicon sequencing.

**Results:**

Alpha diversity tended to be higher in samples from conventional pen birds compared to those from hatch brood birds prior to the day 11 move, but the difference between systems was not observed post-move. Using beta diversity metrics, bacterial community succession appeared delayed in the hatch brood system birds pre-move, but post-move community composition quickly converged with that of the conventional pen birds. This was validated through assessment of significantly different genera between hatch brood system and conventional pen birds, where numbers of significantly different taxa quickly decreased following the move. Some key taxa previously associated with poult performance were delayed in their appearance and relative abundance in hatch brood birds.

**Conclusions:**

Overall, this study demonstrates that the use of isolated hatch brood systems has an impact on the poult gut microbiota, but its impact is resolved quickly once the birds are introduced into a conventional brood environment. Therefore, the benefits of pathogen reduction with hatch brood systems may outweigh negative microbiota impacts due to isolation.

**Supplementary Information:**

The online version contains supplementary material available at 10.1186/s40104-021-00580-4.

## Background

The establishment of a healthy microflora in the developing bird is paramount to poultry production. Several studies have initiated efforts to catalog the commercial turkey gut microbiota [1, 2], to understand its role in health and disease [3], and to identify aspects of the microbiome that correlate with positive performance metrics [4, 5]. While we are gaining broader understanding of what constitutes a healthy microbiota in commercial turkey production, we know very little about the impact of the multitude of variables involved in poultry production on microbiota structure and development. Antibiotic growth promoters have been shown to elicit positive effects on the turkey gut microbiota resulting in enhanced poult performance [6, 7], and the field of probiotics is evolving towards customized approaches in poultry that mimic these positive modulations [8].

Early poult mortality is a significant economic factor in turkey production. The stress of hatching and transporting poults directly to commercial brood barns, often long distances, creates opportunity for mortality to occur due to issues with stress, feed accessibility, and pathogen exposure [9]. At the same time, it is important for turkey poults to acquire a diverse and healthy commensal microbiota as quickly as possible [4]. Brooding birds in specialized isolated units for a period of time prior to placement on commercial brood barns might circumvent many of the problems associated with early feed access and exposure to pathogens. However, it could also delay microbiota development resulting in reduced performance metrics. This study was performed to address the question of delayed microbiota development induced by brooding poults separately from commercial settings.

## Methods

### Birds and experimental design

To identify how a hatch brood turkey poult housing system affects the microbiota of the poult cecum, we divided poults into two housing systems: a novel isolated hatch brood unit and a penned conventional commercial brood facility. The isolated hatch brood system was a proprietary prototype consisting of rectangular plastic crates stacked vertically into columns. The floor of each basket was plastic with holes to allow droppings to pass through onto a cardboard pad placed underneath. The conventional commercial brood facility was a standard brood barn divided into pens using drop-down chain link fencing with brooder guard along the entire length of the bottom to prevent birds and litter from comingling between groups. Pen litter was fresh shavings containing some sunflower seed hull. Both the hatch brood and conventional pen systems allowed birds ad libitum access to standard feed and water.

Poult source was the same for both housing systems. For the hatch brood system, 3200 day-of-hatch turkey Hybrid hens were placed into eight hatch brood columns on day 0. Each crate contained 50 poults and crates were stacked eight high for a total of 400 birds per column. These birds are subsequently referred to as “Hatch-Brood-to-Pen” (HBTP) poults*.* For the conventional brood pen facility, 43,000 day-of-hatch turkey Hybrid hens – subsequently referred to as “Pen” poults – were placed in two separate rooms within the same test barn. One room contained eight pens with 2400 poults in each pen. The other room contained 16 pens; 1700 birds were placed directly into 14 of these pens. The remaining two pens stayed empty until 1500 HBTP birds were moved from the isolated hatch brood system to each pen on day 11 of age (88% density of the conventional brood pens). Cecal content was collected from 10 random poults in each system at eight timepoints (pre-move: days 1, 4, 8, 10 of age; post-move: days 15, 18, 22, 29 of age) for a total of 160 samples.

### Sample collection and processing

All studies were performed on commercial turkeys; therefore, ethical standards for commercial turkey production were followed by the company performing the study. Animals were euthanized using methods approved by the American Veterinary Medical Association. Following euthanasia, birds were promptly and aseptically processed to remove all cecal contents. These samples were hand mixed in sterile bags, subsampled, and stored at − 20 °C prior to processing. DNA was extracted from each sample with the MoBio PowerSoil DNA Isolation Kit (MoBio Laboratories, Carlsbad, CA, USA) following manufacturer’s instructions and stored at − 80 °C. Isolated DNA was used to amplify the V4 region of the 16S rRNA gene using the previously described dual-indexing approach [10]. Library preparation, sample pooling, and paired-end 300-bp sequencing was performed by the University of Minnesota Genomics Center (Minneapolis, MN) on the Illumina MiSeq platform with v3 chemistry. The resulting sequencing reads were demultiplexed using the Illumina MiSeq software.

### Data availability

Raw data from this project is publicly available through the National Center for Biotechnology Information (NCBI) short read archive under BioProject number PRJNA659849.

### Microbial profiling and statistical analyses

Initial quality filtering of sequencing reads was performed using Trim Galore! (v0.6.0) [11], a wrapper script for the software Cutadapt [12] and FastQC [13]. Specifically, bases with a Phred score < Q20 were trimmed from the 3′ end, adaptors and sequences associated with Illumina library preparation were removed using the auto-detect option, and reads < 150 bp were filtered out. Trimmed reads were then processed using the DADA2 pipeline (v1.8.0) [14] within R (v3.6.0) (R Core Team, 2019) following the pipeline’s online tutorial [15]. The SILVA rRNA database (v128) was used to assign taxonomy [16]. The resulting count table of amplicon sequence variants (ASVs) was then filtered accordingly: ASVs were removed if they 1) were classified as chloroplasts, mitochondria, Eukaryota, Archaea, or unknown, 2) had < 10 reads, or 3) occurred in only one sample. Samples with < 10,000 total reads were also removed.

Prior to calculating alpha diversity indices, reads per sample were standardized by rarefying each sample to 11,402 reads, the lowest read count of a sample. ASV richness and the Shannon diversity index were then calculated using the *specnumber* and *diversity* functions in the R package, vegan (v2.5–6) [17]. Nonparametric Wilcoxon rank-sum tests were used to assess differences in alpha diversity between housing systems, with *P*-values adjusted for multiple testing using the Benjamini-Hochberg procedure.

For beta diversity analyses and differential abundance testing, unrarefied data were normalized using cumulative sum scaling (CSS) implemented in the phyloseq package (v1.28.0) [18, 19]. Bray-Curtis dissimilarities and both weighted and unweighted UniFrac distances were calculated with the phyloseq *distance* function and visualized using principal coordinates analysis (PCoA). Euclidean distances between PCoA cluster centroids were calculated with the *dist* function in the package, stats (v3.6.1). To test for differences in the community composition of samples from days and housing systems, PERMANOVAs were performed on dissimilarity matrices using the vegan *adonis* function with 999 permutations. To assess changes in community similarity over time between samples from the same housing system (Pen vs. Pen and HBTP vs. HBTP) and between samples from different housing systems (Pen vs. HBTP), boxplots of collection day by Bray-Curtis dissimilarity were created using the package, ggplot2 (v3.3.2) [20].

Read counts were aggregated by taxonomic level using the phyloseq *tax_glom* function. Lactobacilli are of particular interest within the turkey microbiota and so were further investigated at the species-level. Specifically, all ASVs classified as *Lactobacillus* according to the SILVA rRNA database were aligned to the NCBI nucleotide collection database using blastn [21] with a percent identity of ≥99%. Read counts for ASVs positively identified as a particular species of lactobacilli were then aggregated. When the BLAST results could not distinguish between several closely related lactobacilli species, the read counts for all similar ASVs were aggregated (e.g. *Lactobacillus acidophilus*, *L. crispatus*, and *L. gallinarum*). It should be noted that a reclassification of the genus *Lactobacillus* into 25 novel genera was recently proposed [22]. While we refer to the lactobacilli species by their newly designated taxonomic classifications, for the purposes of continuity with previous turkey gut microbiome literature we also include the traditional names (as listed in the SILVA rRNA database) where appropriate.

Identification of differentially abundant genera and lactobacilli species between systems was conducted using zero-inflated Gaussian mixture models implemented with the *fitZig* function in the R package, metagenomeSeq (v1.26.2) [23]. Genera or species that occurred in < 2 samples for a given comparison were not analyzed. The number of estimated effective samples per genera or species was calculated using the *calculateEffectiveSamples* function and those features with less than the mean number of effective samples in all features were removed. Resulting *P*-values were adjusted for multiple testing with the Benjamini-Hochberg procedure. An alpha value of 0.05 was used for all statistical tests.

## Results

A total of 8,002,173 raw reads were generated from sequencing. Post-quality filtering, a total of 5,011,745 reads remained from cecal content (mean: 31,665 reads/sample, range: 4–62,873) (Additional file 2: Supplementary Table S1). Subsequent filtering of the ASV count table removed 83 ASVs for a final count of 1096 ASVs. Of the 160 samples collected for this study, 139 were retained for downstream analyses.

### Alpha diversity

**Fig. 1:**
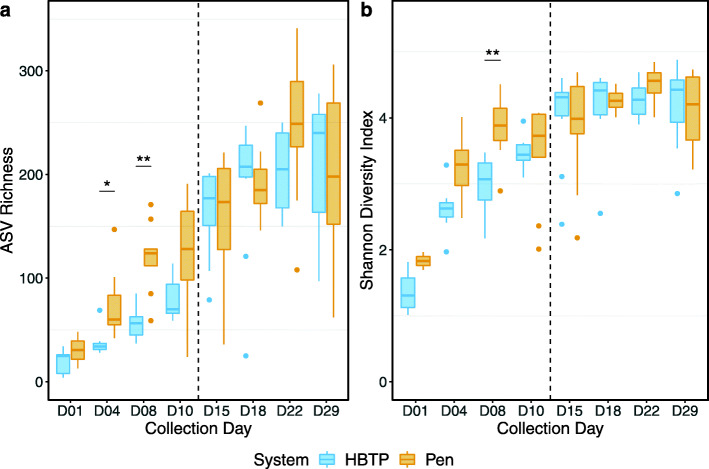
ASV richness (**a**) and the Shannon diversity index (**b**) for each collection day from Pen and HBTP poults. In all box-and-whisker plots, the box spans the 25th–75th percentiles, the line indicates the median, whiskers show minimum and maximum observations, and dots represent outliers. The vertical dashed line between day 10 and day 15 represents when HBTP poults were moved to the conventional pen system. * adjusted *P* ≤ 0.05; ** adjusted *P* ≤ 0.01

Samples exhibited a trend of increased alpha diversity over time (Fig. 1). Further, ASV richness was significantly higher in Pen samples compared to HBTP on both days 4 and 8 (day 4: *W* = 5, adjusted *P* = 0.024; day 8: *W* = 4, adjusted *P* = 0.007) (Fig. 1a; Additional file 2: Supplementary Table S2). Similarly, the Shannon diversity index was higher in Pen samples compared to HBTP on day 8 (*W* = 6, adjusted *P* = 0.005) (Fig. 1b; Additional file 2: Supplementary Table S2). No significant differences in alpha diversity were observed after HBTP poults were moved to the pen system. Interestingly, the largest increase in ASV richness from HBTP samples was observed between the last day pre-move (day 10 mean: 79.4 ASVs) and the first day post-move (day 15 mean: 164.2 ASVs).

### Beta diversity

**Fig. 2:**
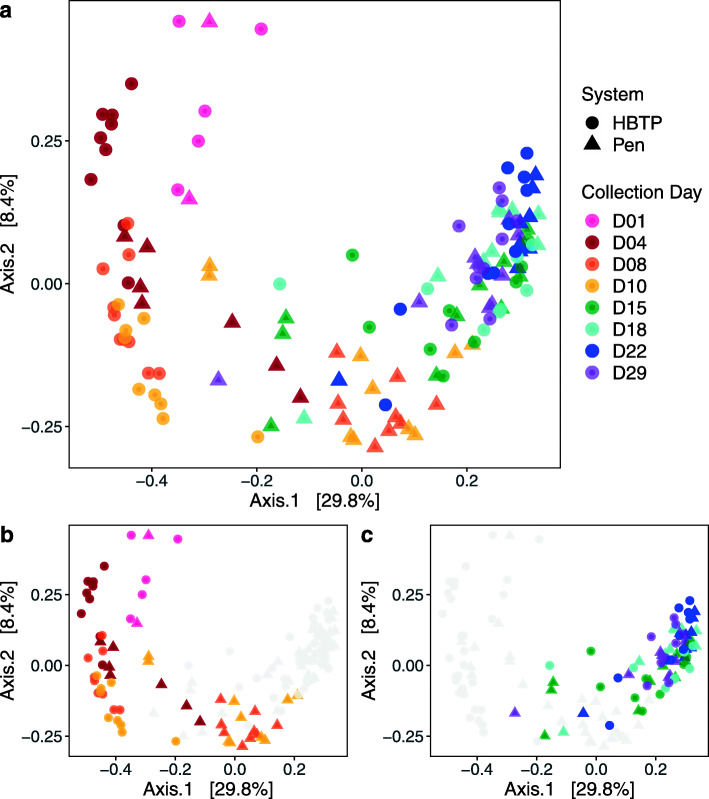
Principal coordinates analysis plots based on Bray-Curtis dissimilarities **a** across all collection days, **b** with only pre-move collection days colored, and **c** with only post-move collection days colored

PCoA using Bray-Curtis dissimilarities showed significant separation between samples collected on different days (PERMANOVA: Pseudo-*F* = 10.17, *R*^2^ = 0.35, *P* ≤ 0.001) (Fig. 2a). Community composition of samples appeared to progress along an age gradient, with greater differences between collection days pre-move (Pseudo-*F* = 6.66, *R*^2^ = 0.26, *P* ≤ 0.001) (Fig. 2b) compared to post-move (Fig. 2c) (Pseudo-*F* = 3.00, *R*^2^ = 0.11, *P* ≤ 0.001). Similar results were also observed using either weighted or unweighted UniFrac distances (Additional file 1: Supplementary Figures S1 and S2).

For all three beta diversity metric PCoAs, pre-move HBTP samples appeared to advance along the age gradient more slowly than Pen samples (Fig. 2b; Additional file 1: Supplementary Figure S1b and S2b). A comparison of Euclidean distances between group centroids revealed that HBTP samples collected on day 4 were on average most similar to day 1 Pen samples, while HBTP day 8 and 10 samples were most similar to day 4 Pen samples (Additional file 1: Supplementary Figure S3). After the move, the community composition of HBTP samples appeared to converge with that of the Pen samples (Fig. 2c, Additional file 1: Supplementary Figure S1c and S2c). Specifically, both HBTP day 15 and day 18 samples were most similar to Pen day 29 samples and both HBTP day 22 and day 29 samples were most similar to Pen day 18 samples (Additional file 1: Supplementary Figure S3).

**Fig. 3:**
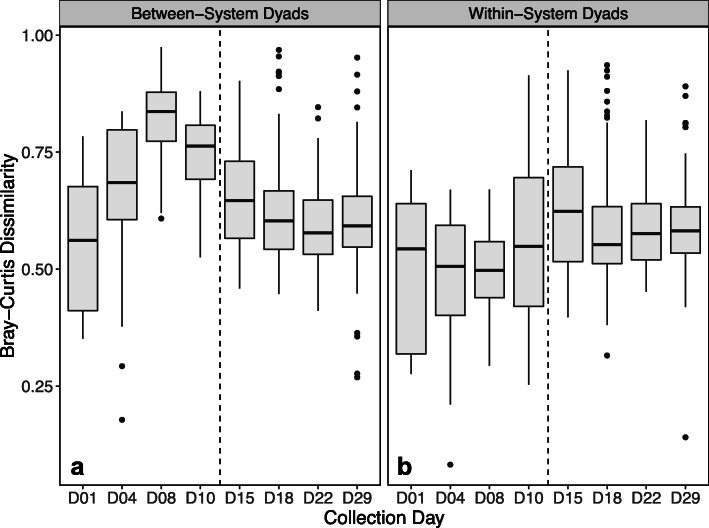
Bray-Curtis dissimilarities between samples from **a** different systems (Pen vs. HBTP) and **b** the same system (Pen vs. Pen and HBTP vs. HBTP). The vertical dashed line between day 10 and day 15 represents when HBTP poults were moved to the conventional pen system

Comparison of between-system sample dyads (Pen vs. HBTP) over collection days similarly showed that average Bray-Curtis dissimilarity between Pen and HBTP samples increased during pre-move days, with maximum dissimilarity on day 8, and subsequently decreased once HBTP poults were moved to the pen system (Fig. 3). A comparable trend was not observed for within-system sample comparisons (Pen vs. Pen and HBTP vs. HBTP), where average dissimilarity remained relatively constant over the collection period (Fig. 3).

### Taxonomic composition and differential abundance testing

**Fig. 4:**
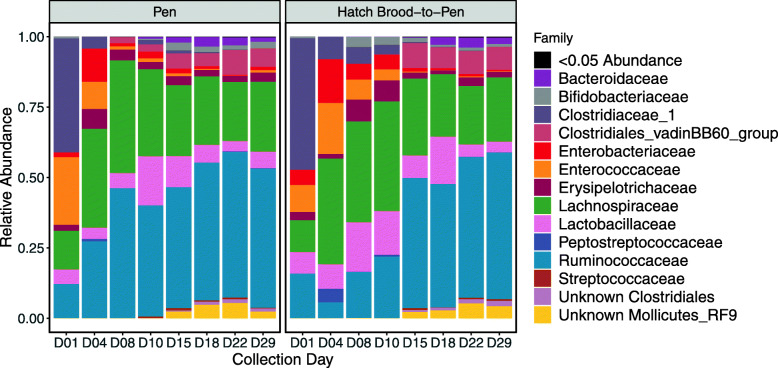
Average relative abundance of the bacterial families in samples from Pen and HBTP systems over time. Families present at < 5% abundance on all collection days are grouped into the “< 0.05 Abundance” category

At the phylum taxonomic level, all samples were dominated by Firmicutes (mean abundance: 89.0%, range: 76.3–100.0% (Additional file 1: Supplementary Figure S4). Within the phylum Firmicutes, the primary families were Lactobacillaceae (Bacilli|Lactobacillales), Enterococcaceae (Bacilli|Lactobacillales), Lachnospiraceae (Clostridia|Clostridiales), and Ruminococcaceae (Clostridia|Clostridiales), except on day 1 when the family Clostridiaceae_1 (Clostridia|Clostridiales) was predominant (Fig. 4). The abundance of Ruminococcaceae and the Clostridiales vadinBB60 group appeared to increase over collection days, while Enterococcaceae decreased. Among other bacterial phyla, there was an increase in members of the class Mollicutes (phylum Tenericutes) and Bacteroidaceae (Bacteroidetes|Bacteroidia|Bacteriodales) starting at day 10. In contrast, abundance of the family Enterobacteriaceae (Proteobacteria|Gammaproteobacteria|Enterobacterales), which includes taxa such as *E. coli* and *Salmonella* spp., decreased over collection days.

**Fig. 5:**
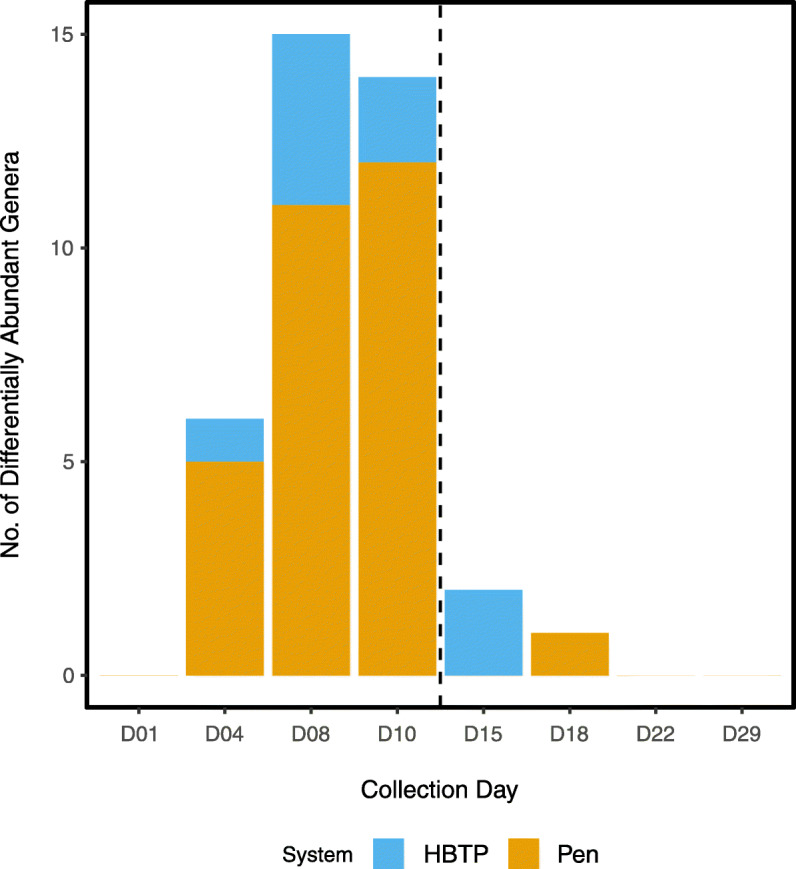
Number of differentially abundant genera in samples stratified by which system (HBTP or Pen) had genera in greater abundance. The vertical dashed line between days 10 and 15 represents when HBTP poults were moved to the conventional pen system

Differential abundance testing between Pen and HBTP samples identified a total of 23 differentially abundant genera on at least one collection day (all adjusted *P* ≤ 0.05) (Additional file 2: Supplementary Table S3). Prior to day 15, the majority of differentially abundant genera were more abundant in Pen samples than HBTP samples (mean number of differentially abundant genera per day: 9.3 in Pen, 2.3 in HBTP) (Fig. 5). Post-move, there was a dramatic decrease in the number of differentially abundant genera and neither Pen nor HBTP samples had consistently more abundant genera. Interestingly, there were no differentially abundant genera between systems on days 22 or 29.

**Fig. 6:**
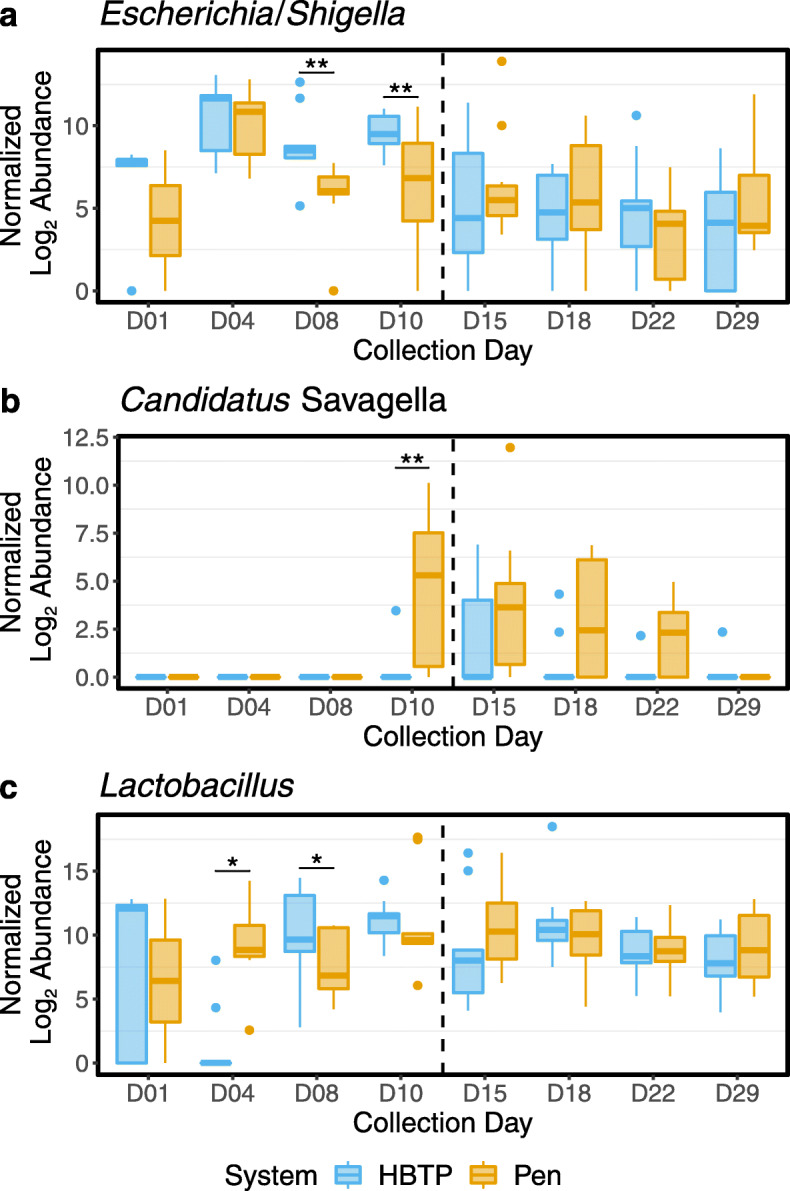
Normalized log_2_ abundance of the genera **a** *Escherichia*/*Shigella*, **b** *Candidatus* Savagella, and **c** *Lactobacillus* (as classified by the SILVA rRNA database) between samples from HBTP and Pen systems over collection days. The vertical dashed line between days 10 and 15 represents when HBTP poults were moved to the conventional pen system. * adjusted *P* ≤ 0.05; ** adjusted *P* ≤ 0.01

Among specific bacterial genera of interest, *Escherichia*/*Shigella* abundance initially increased from day 1 to day 4, but subsequently decreased through day 29 (Fig. 6a). On pre-move days 8 and 10, HBTP samples had a significantly higher abundance of *Escherichia*/*Shigella* compared to Pen samples, but significant differences were absent post-move. Similar to *Escherichia*/*Shigella*, after low levels on days 1, 4, and 8, the abundance of *Candidatus* Savagella, previously known as ‘*Candidatus* Arthromitus’ [24], increased on day 10 for Pen samples and then decreased to day 29 (Fig. 6b). However, *Candidatus* Savagella was absent from the majority of HBTP cecal samples. The abundance of *Lactobacillus* was variable across collection days, with no consistent differential abundance patterns observed between Pen and HBTP samples (Fig. 6c).

Interestingly, at the species level, some lactobacilli did exhibit abundance differences between systems (Additional file 2: Supplementary Table S4). *Ligilactobacillus aviarius* (previously known as *Lactobacillus aviarius*) and *L. acidophilus*/*L. crispatus*/*L. gallinarum* abundances tended to be higher in HBTP samples compared to Pen samples on pre-move collection days, but the differences were largely absent on all post-move days, except for *L. aviarius* on day 15 (Additional file 2: Supplementary Figure S5a, b). Both *Ligilactobacillus salivarius* (previously known as *Lactobacillus salivarius*) and *Limosilactobacillus reuteri* (previously known as *Lactobacillus reuteri*) displayed pre-move patterns opposite to that of *L. aviarius* and *L. acidophilus*/*L. crispatus*/*L. gallinarum*, with higher abundances in Pen samples than HBTP samples (Additional file 2: Supplementary Figure S5c, d). This was particularly evident for *L. salivarius*, where Pen samples had significantly higher abundance compared to HBTP sample on both days 8 and 10. The abundance of *L. johnsonii*/*L. gasseri*/*L. taiwanensis* was more variable throughout the study period, with significantly higher abundance in Pen samples compared to HBTP on day 4 and in HBTP sample compared to Pen on day 10 (Additional file 2: Supplementary Figure S5e).

## Discussion

This study demonstrates that raising turkey poults in isolated hatch brood systems has a measurable impact on their gastrointestinal microbiota, as expected at the outset of these experiments. Overall, patterns were observed that are similar to previously published studies examining the turkey microbiota [1, 3, 4, 6–8, 25], including increasing bacterial diversity as the developing poult ages. Also similar to previous studies, we observed a predictable gradient of bacterial community composition as the bird ages. Finally, key bacterial species were identified that have previously been proposed as microbial biomarkers of turkey gut microbiota succession, including *Escherichia*/*Shigella*, *L. aviarius*, *L. johnsonii*, and *Candidatus* Savagella. These observations reinforce the concept of a predictable succession of bacterial species as the turkey poult develops, strengthening the idea that modulation and support of these key microbes may be beneficial towards development and performance.

We expected differences between the microbiota of HBTP and Pen groups, and this was observed, including delay in bacterial community development in the HBTP group. Intuitively, lack of a diverse source of bacteria in the environment in which a poult is raised will impact and possibly delay the establishment of a diverse microbiota in the gut. However, it was surprising how quickly the HBTP group’s bacterial community converged with those birds in the conventional brood barn in which they entered at 11 days of age. Within 10 days following the movement of poults within this barn, their gut bacterial community compositions were no longer discernibly different. This is encouraging because it suggests that employing these practices to reduce stress and pathogen exposure, while delaying bacterial community development, appears to be quickly resolved following movement to conventional commercial barn environment. While this study did not explicitly examine the mechanisms underlying the shift in HBTP bird microbiota post-move, there was no direct contact between HBTP and Pen birds and so the environment of the conventional brood barn, not the pen birds themselves, was likely the primary source of microbes. Thus, raising birds in an isolated hatch brood system for a period of time prior to placement on conventional brood barns should not be affected by the presence or absence of birds raised in the conventional pens from day-of-hatch.

A number of predicted taxa were identified that were significantly different in their relative abundance between HBTP and Pen groups prior to their move into the conventional brood barn. One of these was the genus *Candidatus* Savagella, previously known as ‘*Candidatus* Arthromitus’ [24], which was nearly absent from HBTP birds prior to movement, but present at relative abundances and ages previously observed in the Pen poults [4]. *Candidatus* Savagella is a segmented, filamentous bacterium whose appearance and relative abundance has been previously shown to highly positively correlate with poult performance [4]. This bacterium is well documented in rodent models as inducing a proinflammatory response in the ileum, which is thought to be involved in priming mucosal immunity for the tolerance of commensal bacteria and targeting of pathogens [25]. The lack of detection of this bacterial species in the HBTP group supports our previous hypothesis that the primary route of acquisition of this bacteria is via its spores present in the environment, which would likely be lacking in an isolated hatch brood system using enhanced disinfection methods, but present in a conventional brood barn. In contrast, *L. aviarius* is another microbial marker that has been observed in multiple studies as being highly positively correlated with bird performance [4, 26]. ASVs classified as *L. aviarius* were not found in any HBTP sample until after the movement of birds into the conventional pens. At the same time, ASVs classified as other lactobacilli species (such as *L. salivarius* and *L. reuteri*) tended to display higher relative abundance in Pen versus HBTP groups during the same sampling period. Because *L. aviarius* appears to be a highly host-adapted species, we speculate that these bacteria are either acquired during hatch or possibly vertically transmitted, and as such, they colonized both groups early in life. Again, because the hatch brood environment likely contains fewer exogenous lactobacilli species than a traditional brood barn, this would create a niche for elevated *L. aviarius* colonization as observed in HBTP poults. Regardless, it raises questions about precisely which bacterial species may be vertically transferred from hen to poult, and this warrants additional study. Furthermore, the lack of some key bacterial species associated with poult performance suggests that targeted use of probiotics in hatch-brood settings may aid in the diversification and development of the turkey poult microbiota.

There were some limitations in this study. First, only one biological replicate was performed. Additional replicates will be necessary to validate that our observations are reproducible in similar and diverse poultry production settings. Second, no birds remained in the hatch brood system throughout the experiment. Without this control group it is unclear whether the microbiota of the HBTP birds would still have converged with the Pen bird microbiota had they remained in the hatch brood system. That said, the hatch brood units used in this study were only designed for early brood use and thus could not effectively house the growing poults throughout the entirety of the experiment. Third, pen density was not controlled for between Pen birds and post-move HBTP birds. Ideally, the pen densities would have been the same for both experimental groups. However, given there were few microbial differences between groups post-move, the small difference in density likely did not have a large impact on our principal findings. Fourth, performance parameters were not measured in this study. It will be important in future studies to confirm that hatch brood rearing has no significant impact on performance. Additionally, specific pathogens were not assessed. If the assumption is that hatch-brood systems benefit poultry production by reducing the introduction of specific pathogens, this will need to be confirmed.

## Conclusions

This study demonstrates that raising turkey poults in an isolated hatch brood system results in differential succession of their gut microbiota, compared to rearing in conventional brood barn facilities. However, the differences in microbiota succession are quickly alleviated upon poult introduction to conventional brood pen environments. This indicates that the initial use of hatch brood systems prior to time in conventional brood barns may not significantly impact the overall development of the turkey microbiota towards a healthy and productive animal.

## Supplementary Information

**Additional file 1.** Supplementary Figures S1-S5.

**Additional file 2.** Supplementary Tables S1-S4.

## Data Availability

The dataset supporting the conclusions of this article is available in the NCBI short read archive under BioProject number PRJNA659849.

## Abbreviations

ASV: Amplicon sequence variant
CSS: Cumulative sum scaling
HBTP: Hatch brood-to-pen
NCBI: National Center for Biotechnology Information
PCoA: Principal coordinate analysis
