# Supplementary material for: Convergence of the turkey gut microbiota following cohabitation under commercial settings
Source: J Anim Sci Biotechnol. 2021 May 5;12:59. doi: 10.1186/s40104-021-00580-4 (PMC8097982; doi:10.1186/s40104-021-00580-4)
Supplement: Supplementary file 1 — Additional file 1. Supplementary Figures S1-S5. [file 40104_2021_580_MOESM1_ESM.docx]

**SUPPLEMENTAL FIGURES**

**Convergence of the turkey gut microbiota following cohabitation**

**under commercial settings**

Elizabeth A. Miller^1,†^, Brittanie Winfield^1,†^, Bonnie P. Weber^1^, Cristian Flores-Figueroa^2^, Jeannette Munoz-Aguayo^2^, Jared Huisinga^3^, Timothy J. Johnson^1,2,*^

^1^University of Minnesota, Department of Veterinary and Biomedical Sciences, Saint Paul, MN, USA

^2^University of Minnesota, Mid-Central Research and Outreach Center, Willmar, MN, USA

^3^Life Science Innovations, Willmar, MN, USA

*Corresponding author. Email address: tjj@umn.edu

^†^Co-first authors.

**Figure S1.** Principle coordinates analysis plots based on weighted UniFrac distances for samples across (**a**) all collection days, (**b**) only pre-move collection days colored, and (**c**) only post-move collection days colored. Circles indicate Hatch Brood-to-Pen (HBTP) samples, triangles indicate Pen samples, and shapes are colored by collection day.

**Figure S2.** Principle coordinates analysis plots based on unweighted UniFrac distances for samples across (**a**) all collection days, (**b**) only pre-move collection days colored, and (**c**) only post-move collection days colored. Circles indicate Hatch Brood-to-Pen (HBTP) samples, triangles indicate Pen samples, and shapes are colored by collection day.

**Figure S3.** Principle coordinates analysis plots based on Bray-Curtis dissimilarities depicting system-day group centroids for samples. Dashed arrows between points indicate the closest Pen-day group to each HBTP-day group based on Euclidean distance.

**Figure S4.** Relative abundance of the bacterial phyla in samples from Pen and HBTP systems over time. Phyla present at < 1% abundance on all collection days are grouped into the “<0.01 Abundance” category.

(Figure S5 continued next page)

**Figure S5.** Normalized log2 abundances of the lactobacilli species (**a**) *Lactobacillus* *acidophilus*/*L. crispatus*/*L. gallinarum*, (**b**) *Ligilactobacillus* [*Lactobacillus*] *aviarius*, (**c**) *Ligilactobacillus* [*Lactobacillus*] *salivarius*, (**d**) *Limosilactobacillus* [*Lactobacillus*] *reuteri*, and (**e**) *L. johnsonii*/*L. gasseri*/*L. taiwanensis* between samples from HBTP and Pen systems over collection days. Plots are box-and-whisker plots, where the box spans the 25th–75th percentiles, the line indicates the median, whiskers show minimum and maximum observations, and dots represent outliers. The vertical dashed line between days 10 and 15 represents when HBTP poults were moved to the conventional pen system. Statistically significant differences between systems are denoted by asterisks below the *x*-axis labels. A reclassification of *Lactobacillus* into 25 novel genera was recently proposed by Zheng et al. 2020 (Int J Syst Evol Microbiol 70: 2782-2858). Traditional nomenclature is included in brackets where applicable. * adjusted *P* ≤ 0.05; ** adjusted *P* ≤ 0.01; *** adjusted *P* ≤ 0.001
